# Paroxysmal Idiopathic Abdominal Pulsations

**Published:** 1888-06

**Authors:** A. H. McCord

**Affiliations:** Rusk, Texas


### PAROXYSMAL IDIOPATHIC ABDOMINAL PULSATION.

*By A. H. McCord, M. D., Rusk, Texas*

Read befre Cherokee County Medical Society, and by vote of Society sent for publication in Daniel's Texas Medical Journal.

On 27th August, 1880, was called to see J. K., mail agent, about 55; found him suffering with intense pain, located in the rigion of right kidney and along the course of the right ureter. Pulse 100, some fever. Urine scanty and high colored. Some difficulty and pain on micturition. He told me he had passed some small "gravels." Used hot hip-baths, anodynes and alkaline diuretics,which afforded some relief. He slept well during the night. August 28, suffered some all day with pain in lumbar region; pulse about 100 during the day; still some fever; at 8 p. m. fever subsided, pain entirely relieved; slept well at night, was cheerful next morning, 29th; was able to be at the breakfast table, to take his meal, also at dinner, ate hearty. At 4 p. m. pain returned in right lumbar region, but soon began to radiate to the right hypochondriac and sub-scapular regions. No fever—no acceleration of pulse; patient began to notice abdominal "throbbing," as he termed it. On examination I found this pulsation synchronous with the heart beat. The pulsation was very violent and disagreeable to the patient. Used anodyne and counter-irritants and about 8 p. m. patient again found relief. Slept well at night, was cheerful in the morning of the 30th. No abdominal pulsation. At 4 p. m. was again seized with very acute pains as on the previous day; no fever, no pulse rise, but a return of the aortic pulsation, more disagreeable than ever. Used stimulants and counter-irritation along the spinal column with some relief to patient. At 8 p. m. pain again ceased and pulsation stopped and he was free from any disturbance, except a sense of general weakness, until about 4 p. m. of the 31. Anticipating another attack at that hour from its tendency to periodicity, I ordered quinine and stimulants to be given freely, which had the good effect of breaking the force of the attack. I again ordered quinine and stimulants to be given September 1, which still further lightened the attack, which still persisted in coming on about 4 p. m. and quitting about 8 p. m. Again I ordered the same course of medication to be followed for

September 2, which controlled the praroxysms and the patient was discharged.

Mr. K. lived in a very malarious district. He had had an attack of remittent fever some two weeks previous to the aortic vaso-motor disturbance. He was of a nervous temperament. Hygienic surroundings not good. Diet coarse. I present this case because of the scarcity of literature on the subject. So far as I know our American authors make but a reference to it.

The disease in question is evidently a local neurosis, due to a perturbed innervation in the vaso-motor nerves of the abdominal aorta—a plexus of the ganglionic system run riot—a local insanity, as it were, in the aortic plexus of the sympathetic nervous system. With reference to the prognosis, so far as known, no case has ended in death; but Macario says: "Long existence of the disease may lead to metal derangement—even to suicide. Hence, this affection, when left to itfelf, without prompt judicial medical interference, is of sufficient moment to make us solicitious about its ultimate grave tendencies. I claim this case to be one of *the* peculiar manifestations of malaria.

